# Supplementary figures and images for: Astrocyte-Secreted Lcn2 Modulates Dendritic Spine Morphology
Source: Cells. 2025 Jan 21;14(3):159. doi: 10.3390/cells14030159 (PMC11817088; doi:10.3390/cells14030159)

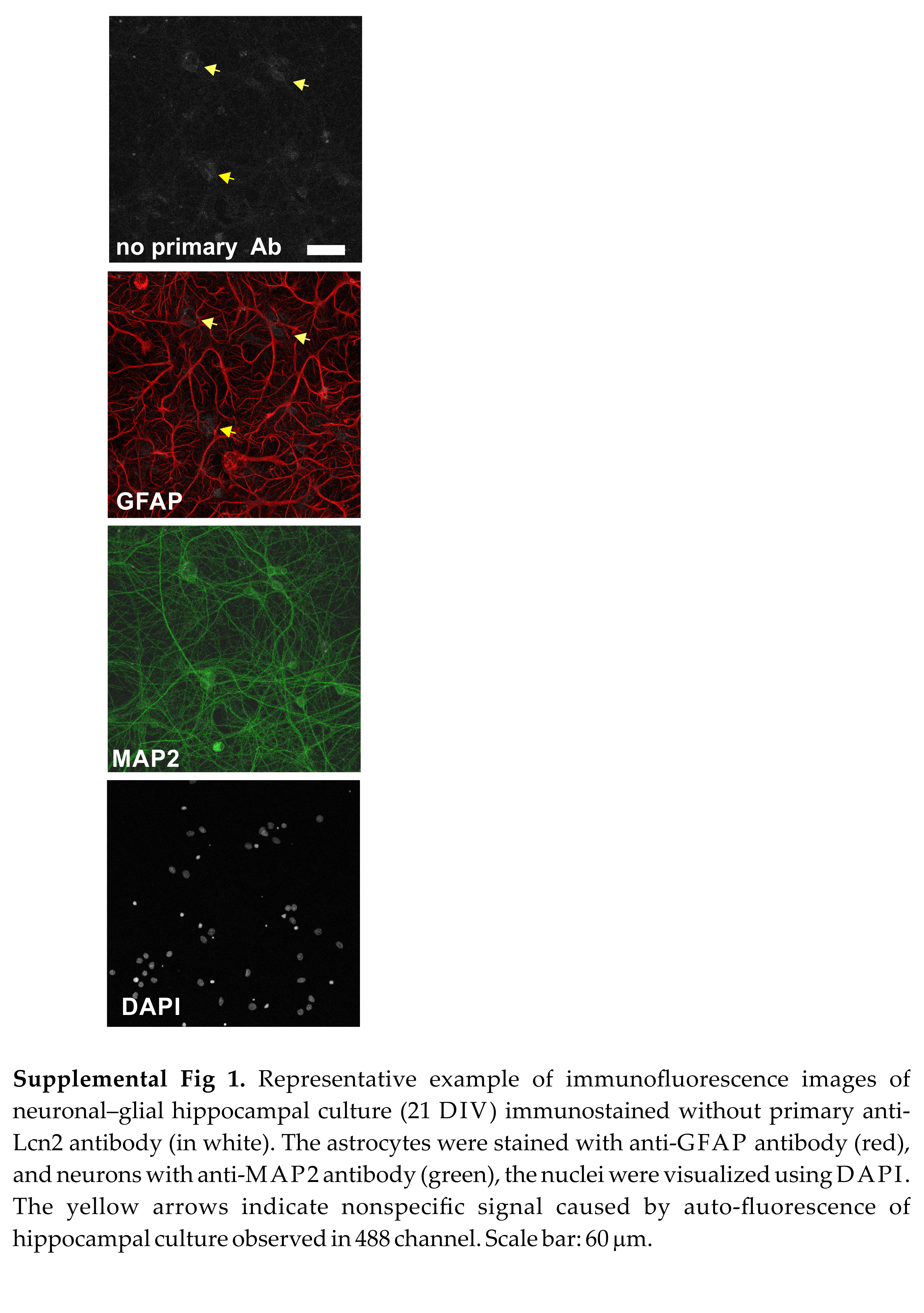

Supplement: Supplementary file 1 [file cells-14-00159-s001.zip › Supl_1.tif]

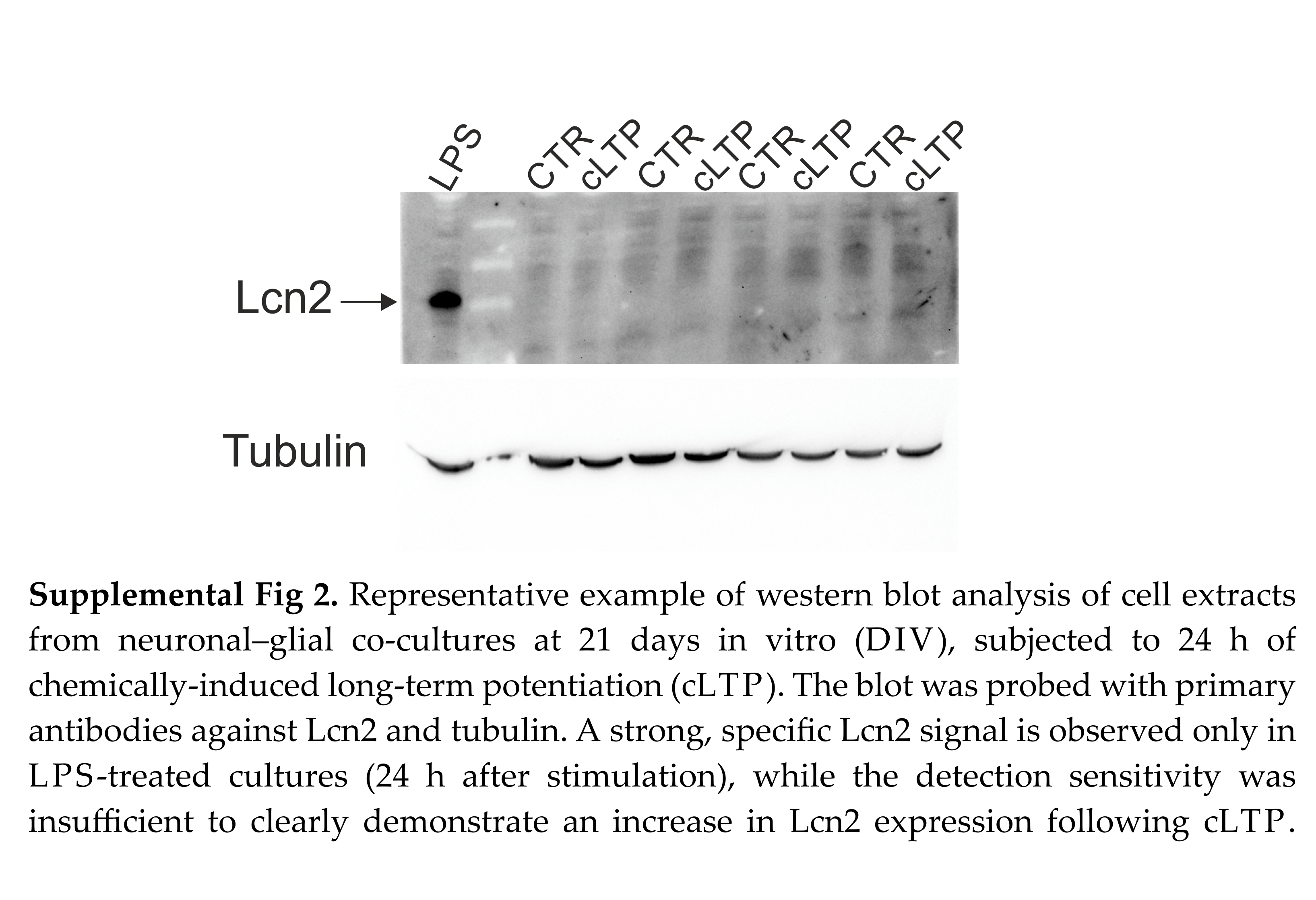

Supplement: Supplementary file 1 [file cells-14-00159-s001.zip › Supl_2.tif]
